# All-Solid-State Lithium-Ion Batteries with Oxide/Sulfide Composite Electrolytes

**DOI:** 10.3390/ma14081998

**Published:** 2021-04-16

**Authors:** Young Seon Park, Jae Min Lee, Eun Jeong Yi, Ji-Woong Moon, Haejin Hwang

**Affiliations:** 1 Department of Materials Science & Engineering, Inha University, Incheon 22212, Korea; pys3621@inha.edu (Y.S.P.); 22201275@inha.edu (J.M.L.); inha0326@inha.ac.kr (E.J.Y.); 2 Battery Materials Research Center, Research Institute of Industrial Science and Technology, Pohang 37673, Korea; jwmoon@rist.re.kr

**Keywords:** all-solid-state lithium-ion batteries, composite solid electrolytes, sulfides, argyrodite, garnet, lithium-ion conductivity

## Abstract

Li_6.3_La_3_Zr_1.65_W_0.35_O_12_ (LLZO)-Li_6_PS_5_Cl (LPSC) composite electrolytes and all-solid-state cells containing LLZO-LPSC were fabricated by cold pressing at room temperature. The LPSC:LLZO ratio was varied, and the microstructure, ionic conductivity, and electrochemical performance of the corresponding composite electrolytes were investigated; the ionic conductivity of the composite electrolytes was three or four orders of magnitude higher than that of LLZO. The high conductivity of the composite electrolytes was attributed to the enhanced relative density and the rule of mixture for soft LPSC particles with high lithium-ion conductivity (~10^−4^ S·cm^−1^). The specific capacities of all-solid-state cells (ASSCs) consisting of a LiNi_0.8_Co_0.1_Mn_0.1_O_2_ (NCM811) cathode and the composite electrolytes of LLZO:LPSC = 7:3 and 6:4 were 163 and 167 mAh·g^−1^, respectively, at 0.1 C and room temperature. Moreover, the charge–discharge curves of the ASSCs with the composite electrolytes revealed that a good interfacial contact was successfully formed between the NCM811 cathode and the LLZO-LPSC composite electrolyte.

## 1. Introduction

The improvement of the safety and energy density of lithium-ion batteries is important for their application as a power source in large-scale devices such as electric vehicles [1]. Hence, the development of all-solid-state lithium batteries (ASSLBs) with incombustible inorganic solid electrolytes is highly anticipated. ASSLBs are considered to be next-generation batteries because they can store more energy and are safer to operate than lithium-ion batteries (LIBs) [2]. In addition, ASSLBs have a lower number of battery components and require less packing space than LIBs, thereby reducing the weight and volume of battery-based devices [3].

Garnet-structured Li_7_La_3_Zr_2_O_12_ (LLZO) is a promising solid electrolyte material because it exhibits higher ionic conductivity (in the order of 10^−4^ to 10^−3^ S·cm^−1^) and higher electrochemical stability against lithium metal [4,5,6]. However, LLZO requires high-temperature sintering to achieve high relative density (>95%). NASICON-type LiTi_2_(PO_4_)_3_ (LTP), LiGe_2_(PO_4_)_3_ (LGP), and related ceramics are also typical lithium-ion conductors with ionic conductivities as high as 10^−4^ S·cm^−1^ at room temperature [7]. Among these materials, Al-doped LTP exhibits the highest room-temperature conductivity (in the order of 10^−3^ S·cm^−1^) and is known to be chemically stable in humid air or carbon dioxide. However, LTPs and LGPs suffer from higher resistance at the grain boundaries and are unstable against lithium-metal anodes [8,9].

In contrast, sulfide-based solid electrolytes such as Li_2_S-P_2_S_5_ (LPS)-based glass or glass ceramics and Li_6_PS_5_X (X = Cl, Br, I) with an argyrodite crystal structure are superionic conductors with high electrochemical stability and ionic conductivity [10]. Owing to their negligible grain-boundary resistance, sulfide-based electrolytes exhibit excellent conductivity even under cold-pressing conditions. Ionic conductivities of 2.2 × 10^−3^ and 1.3 × 10^−3^ S·cm^−1^ have been achieved for Li_7_P_3_S_11_ and Li_3_PS_4_ glass ceramics, respectively [11,12]. Recently, Jung et al. reported that cold-pressed Li_5.5_PS_4.5_C_l1.5_ exhibited an ionic conductivity of 10.2 × 10^−3^ S·cm^−1^ at room temperature [13]. However, the main drawback of these sulfide electrolytes is that they must be handled in an inert gas atmosphere because of their low chemical stability in air. The hydrolysis of the sulfides in such electrolytes by water molecules in air generates H_2_S [14], which is detrimental to battery operation and life.

Recently, composite electrolytes based on oxides/sulfides and halides/sulfides that are capable of realizing room-temperature cell fabrication (due to the presence of sulfides and halides) and that exhibit good chemical and electrochemical stability (due to the presence of oxides) have been developed [15,16,17,18]. An oxide/sulfide composite electrolyte for all-solid-state batteries was investigated by Rangasamy et al. [15]. They addressed that the composite electrolyte can combine the advantages of LPS and LLZO. Moreover, halide/sulfide composite systems were proposed by El Kharbachi et al. [16,17]. This report outlines the halide and sulfide interaction that is responsible for the enhanced lithium-ion conductivity.

In this study, garnet-type oxide LLZO-based composite electrolytes containing argyrodite Li_6_PS_5_Cl (LPSC) particles were proposed. The presence of sulfide-facilitated lithium-ion transport in the composite electrolyte, leading to high room-temperature lithium-ion conductivity. The effect of sulfide on the microstructure and ionic conductivity of the composite electrolytes was investigated. All-solid-state cells (ASSCs) consisting of a lithium nickel cobalt manganese oxide (NCM)/LPSC/carbon black cathode, an oxide (LLZO)-sulfide (LPSC) composite electrolyte, and an indium lithium alloy anode were fabricated. The sulfide:oxide ratio was varied, and the performance of the corresponding ASSCs was evaluated.

## 2. Materials and Methods

The composite electrolytes were fabricated from LLZO and LPSC powders. Tungsten-doped LLZO powder (99.9%) with a nominal composition of Li_6.3_La_3_Zr_1.65_W_0.35_O_12_ was purchased from Ampcera Inc. (Milpitas, CA, USA). The average particle size (D50) of the W-LLZO was 10 μm. The LPSC sulfide powder was mechanochemically synthesized by mixing Li_2_S (99.9%, Alfa Aesar, Haverhill, MS, USA), P_2_S_5_ (99.9%, Alfa Aesar, Haverhill, MS, USA), and LiCl (99.9%, Alfa Aesar, Haverhill, MS, USA) powders in a planetary mill. High-energy milling was carried out using 2 g of the powder mixture with zirconia balls (∅10, ∅5, ∅3, and ∅2 mm) in a zirconia jar (80 mL). A Pulverisette 6 planetary mill (Fritsch, Idar-Oberstein, Germany) operating at a rotational speed of 500 rpm was used to mill the powder mixture for 16 h (30 min of milling followed by 30 min resting). The powder samples were then annealed at 550 °C in a tube furnace for 8 h. The annealing process was conducted in an argon atmosphere.

The phase identification of the synthesized LPSC powder and composite electrolyte samples was performed through X-ray diffraction (XRD) analysis (RU-200B, Rigaku Co., Ltd., Tokyo, Japan) with Ni-filtered Cu-Kα radiation. The bulk density was calculated by measuring the weight and volume of composite electrolyte pellets; the theoretical density of the composite electrolyte was calculated using the rule of mixture equation. Based on the calculated bulk and theoretical densities, the relative density was evaluated. The microstructure was examined through field-emission scanning electron microscopy (FE-SEM; JSM-6700F, JEOL, Tokyo, Japan).

Moreover, the ionic conductivities of the pelletized composite electrolyte samples were measured. Disk-type pellet samples with a diameter of 10 mm and a thickness of 0.5 mm were cold-pressed at 300 MPa. The alternating current (AC) impedance spectra were obtained from the composite electrolyte pellet with two stainless-steel rods acting as current collectors under open-circuit conditions with an excitation potential of 20 mV over a frequency range of 1 MHz to 0.01 Hz using an impedance analyzer (SP-300, Biologic, France). The voltage and current resolution of the impedance analyzer were 50 μV and 10 nA, respectively. Two wires from the two stainless-steel electrodes were connected to the working and counter terminals of the impedance analyzer. The ionic conductivity, *σ*, was calculated using the equation: *σ* = *t*/RA, where R is the total resistance of the composite electrolyte, *t* is the sample thickness, and A is the area of the composite electrolyte.

ASSCs (∅10 mm), consisting of lithium nickel cobalt manganese oxide, LiNi_0.8_Co_0.1_Mn_0.1_O_2_ (NCM811)/LPSC/super P as the cathode, LLZO/LPSC as the composite electrolyte, and an indium (In)-lithium (Li) alloy foil as the anode, were assembled by cold pressing at 300 MPa. The NCM811 powder was synthesized in the laboratory by precipitation. To fabricate the cathode, NCM811, LPSC, and super P were mixed in a mortar for 30 min at a weight ratio of 70:29:1. The In–Li alloy foil was prepared in the laboratory from In (127 μm, Alfa Aesar, Haverhill, MS, USA) and Li/Cu (50 μm, Honjo Metal Co. Ltd., Higashiosaka, Japan) foils. ASSCs were fabricated using a 3-mol%-yttria-stabilized tetragonal zirconia polycrystal (3Y-TZP) mold (∅10 mm) with stainless-steel rods (∅10 mm). First, the LLZO/LPSC composite powder was pressed at 300 MPa to form a composite electrolyte pellet. The cathode composite powder was then pressed on one side of the composite electrolyte pellet at 300 MPa. Finally, the In and Li/Cu foils were attached to the other side of the composite electrolyte pellet by applying a pressure of 10 MPa.

The charge–discharge behavior of the ASSCs was studied by using a battery test system (SP-300, Biologic, Seyssinet-Pariset, France) with a cutoff voltage of 1.9–3.6 V (vs. Li–In) at room temperature (25 °C). The current density was set to 0.535 mA·cm^−2^. Charging and discharging were carried out in constant current (CC)–constant voltage and CC modes, respectively.

## 3. Results and Discussion

**Figure 1:**
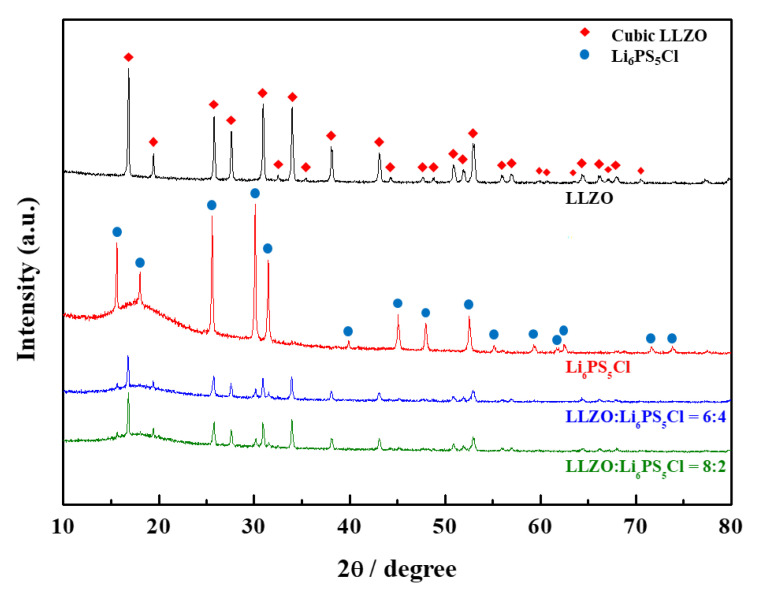
XRD patterns for LLZO, LLZO-LPSC composite, and LPSC electrolytes.

Figure 1 shows the XRD patterns of the LLZO-LPSC composite electrolyte. The XRD patterns of commercially available W-doped LLZO and the synthesized LPSC powders are also shown in Figure 1. In the XRD pattern of the LPSC powder sample, peaks identical to those observed for the standard LPSC were observed [19]. There were no peaks corresponding to the secondary phases or unreacted starting materials such as Li_2_S and LiCl. Mechanical milling (planetary mill) and subsequent annealing at 550 °C produced single-phase LPSC with high crystallinity and no impurity phases. In addition, the XRD peaks of the W-doped LLZO powder indicated that it had a cubic-type garnet structure.

Furthermore, the XRD patterns of the composite electrolyte exhibited the characteristic peaks of cubic LLZO and LPSC crystal structures. There were no peaks of unwanted reaction phases between LLZO and LPSC in the composite electrolyte samples after mechanical mixing and cold pressing at 300 MPa. The intensity of the peak corresponding to the LPSC phase gradually increased with an increase in the LPSC:LLZO ratio. This result indicates that LLZO does not react with LPSC during the fabrication of the composite electrolyte pellet samples.

**Figure 2:**
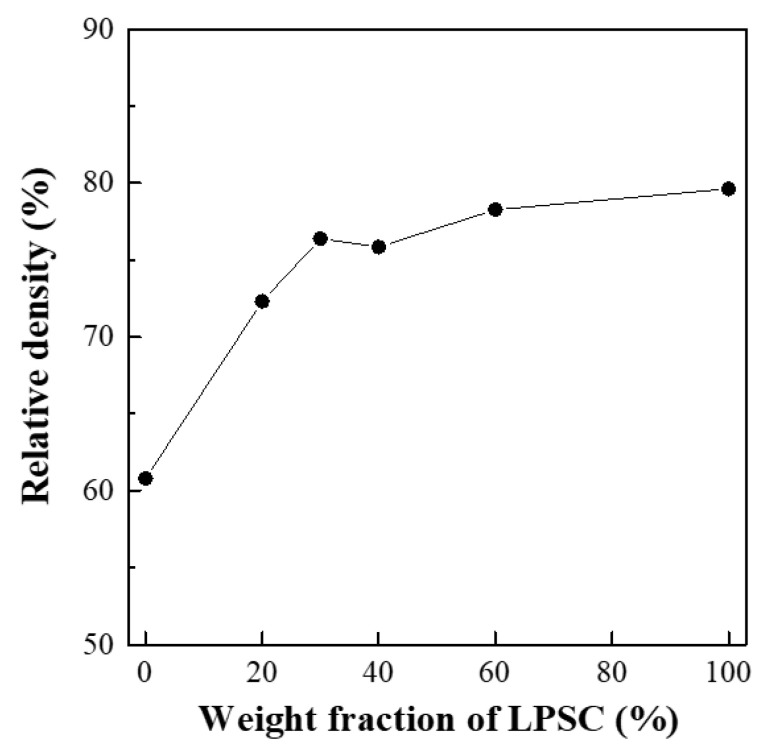
Relative densities of LLZO-LPSC composite electrolytes.

**Figure 3:**
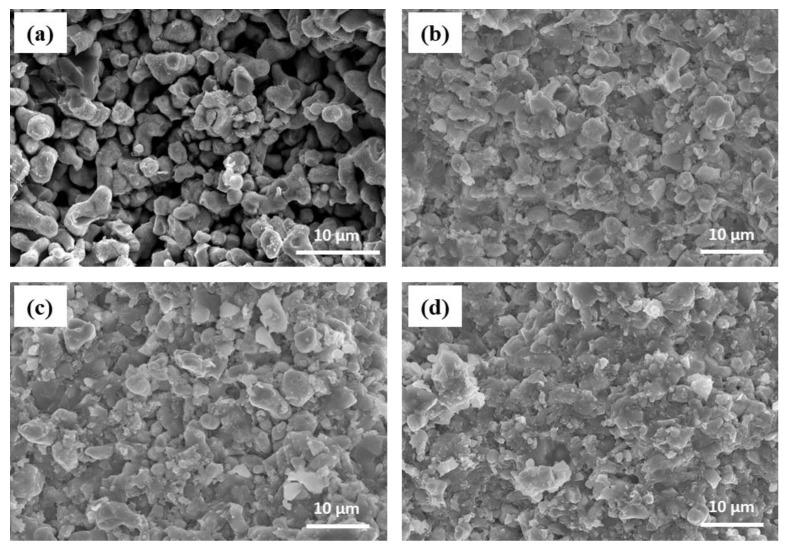
Cross-sectional SEM images of solid electrolytes of (**a**) LLZO (**b**) and at LLZO:LPSC = 8:2, (**c**) LLZO:LPSC = 6:4, and (**d**) LLZO:LPSC = 4:6.

Figure 2 shows the relative densities of the composite electrolyte pellet samples as a function of the weight fraction of LPSC. The theoretical densities of LLZO and LPSC were 5.098 and 1.860 g·cm^−3^, respectively. The relative density of the LLZO pellets was estimated to be 60.8%, while it increased considerably to 72.3% at an LLZO:LPSC ratio of 8:2. Thereafter, it increased slightly with the further addition of LPSC. The soft nature of the LPSC particles enhanced the densification of the LLZO electrolyte pellets upon cold pressing. Cross-sectional SEM images of LLZO and the composite electrolyte pellet samples are shown in Figure 3. The LLZO pellet was highly porous (39% porosity). In contrast, as can be seen in Figure 3b, a dense microstructure was observed in the composite electrolyte pellet samples (LLZO:LPSC = 8:2). With a further increase in the weight fraction of LPSC, no significant microstructural change was observed in the composite electrolyte pellet samples (LLZO:LPSC = 6:4 and 4:6). This is in good agreement with the relative density results shown in Figure 2.

**Figure 4:**
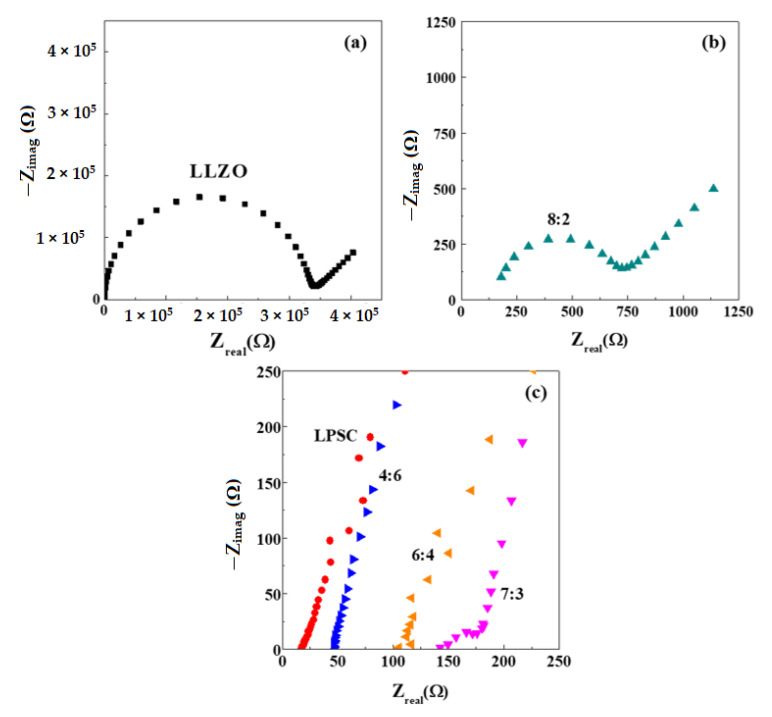
Impedance spectra of symmetrical cells with solid electrolytes (**a**) of LLZO, at (**b**) LLZO:LPSC = 8:2, at (**c**) LLZO:LPSC = 7:3, 6:4, 4:6, and of LPSC.

Figure 4 shows the AC impedance spectra of symmetrical cells consisting of an LLZO-LPSC composite electrolyte and two stainless-steel blocking electrodes measured at 25 °C. For comparison, the AC impedance spectra of the LLZO and LPSC pellets are shown. The spectrum of the LLZO pellet showed a large semicircle at high frequencies, followed by a low-frequency spike, which is typically observed in LLZO symmetrical cells with blocking electrodes [20,21]. The high-frequency semicircle can be divided into two semicircles that correspond to the bulk and grain-boundary resistances [5,22]. In some cases, a depressed semicircle can also be observed [6,23], depending on the density (porosity) or grain size of the sintered electrolyte sample or the measuring temperature.

The impedance spectra of the LLZO pellet could not be well-resolved into bulk and grain boundaries because large amounts of pores were present in the LLZO pellet. This is because it is impossible to obtain a dense LLZO pellet at room temperature by cold pressing. The impedance spectra of the LLZO:LPSC = 8:2 and 7:3 composite electrolyte pellets were similar to that of the LLZO pellet, i.e., a semicircle and a spike. However, the semicircle (bulk and grain-boundary resistances) of the composite electrolyte samples decreased significantly as the LPSC ratio increased, suggesting that the relative density of the composite electrolyte was enhanced by the addition of LPSC particles. LPSC is softer than LLZO and exhibits plastic deformation, which makes it easy to fabricate a densely packed composite electrolyte by cold pressing [24,25].

The impedance spectra of the LLZO:LPSC = 6:4 and 4:6 composite electrolyte pellets were similar to that of the LPSC pellet. While a spike (straight line) was observed, the semicircle in the impedance spectra disappeared, indicating an extremely low grain-boundary resistance [26,27]. These results suggest that the lithium-ion transport in the LLZO:LPSC = 6:4 and 4:6 composite electrolytes is mainly governed by the LPSC weight fraction. The ionic conductivity at room temperature can be derived from the total resistance that is deduced from the intersection of the spike with the real axis at the lower frequency side. The ionic conductivities of the composite electrolytes are listed in Table 1. The conductivity of the LLZO electrolyte pellet was 1.40 × 10^−7^ S·cm^−1^. This value is three or four orders of magnitude lower than that of a fully densified LLZO ceramic. However, this is not surprising because the relative density of the LLZO electrolyte pellet, obtained by cold pressing at room temperature, was as low as 60.8%.

The ionic conductivity of the LLZO:LPSC = 8:2 composite electrolyte was almost three orders of magnitude higher than that of the LLZO electrolyte. This result indicates that compositing with LPSC particles is an effective technique to increase the ionic conductivity of the porous LLZO electrolyte. The high ionic conductivity observed in composite electrolytes is attributable to two factors: the considerably enhanced density of the LLZO:LPSC = 8:2 composite electrolyte and the rule of the mixture effect. Table 1 indicates that the conductivity of the LPSC electrolyte was 2.92 × 10^−3^ S·cm^−1^. Although the ionic conductivity increased upon the addition of LPSC, this increase was not remarkable.

Rangasamy et al. [15] demonstrated that the ionic conductivity of composite electrolytes increases with the sulfide weight fraction, exhibiting a maximum at an oxide (LLZO):sulfide (β-Li_3_PS_4_, LPS) ratio of 3:7, and then decreasing with the addition of sulfide. The authors explained the maximum conductivity achieved at oxide:sulfide = 3:7 in terms of the space-charge effect that forms at the interface of the LLZO and LPS particles. El Kharbachi et al. prepared a halide/sulfide composite electrolyte with a composition of 50Li(BH_4_)_0.75_I_0.25_:50(Li_2_S)_0.75_(P2S5)_0.25_ = 1:2; this electrolyte exhibited the highest lithium-ion conductivity (~10^−3^ S·cm^−1^) and the lowest activation energy (0.3 eV) at room temperature [16]. The improvement of lithium-ion conduction was attributed to the structural modification that occurred due to the incorporation of lithium halide (LI) into the sulfide structure [17].

In this study, the sulfide electrolyte was argyrodite (LPSC), which has a much higher ionic conductivity (~10^−3^ S·cm^−1^) than that of LLZO (10^−7^ S·cm^−1^). In addition, the relative density of the composite electrolytes increased significantly with respect to the LPSC weight fraction. Thus, the space-charge effect or possible interaction between LLZO and LPSC may be counteracted by the increase in the highly conductive and dense LPSC phase.

**Figure 5:**
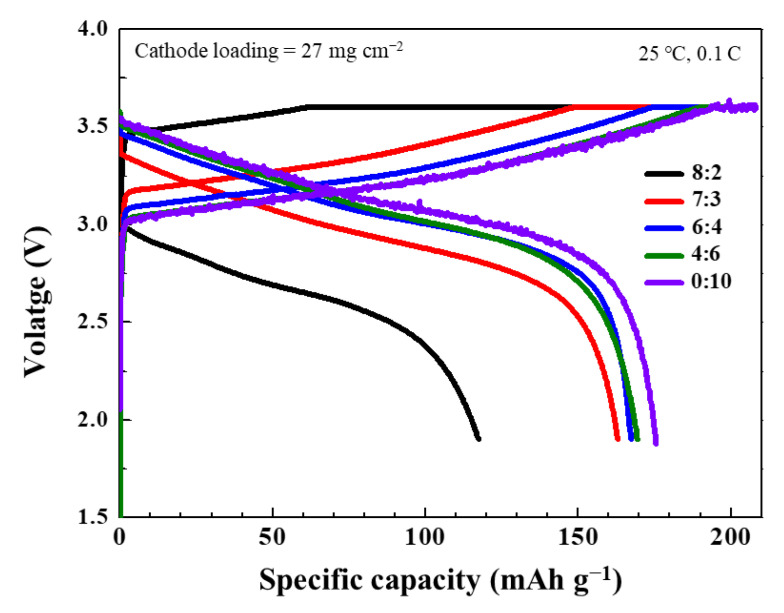
Charge–discharge profiles of the first cycle at 0.1 C of ASSCs with composite electrolytes with different LLZO:LPSC ratios.

Figure 5 shows the charge–discharge curves of the ASSCs with the composite electrolytes in the first cycle. For comparison, the charge–discharge curves of the cells with the LPSC electrolyte are shown in Figure 5. In the case of the cell with the LLZO electrolyte, it is impossible to obtain the charge–discharge curve because of high electrolyte resistance, which is ascribed to the extremely low ionic conductivity of the LLZO electrolyte (1.4 × 10^−7^ S·cm^−1^). The cell with the LLZO:LPSC = 8:2 composite electrolyte exhibited discharge capacity of 118 mAh·g^−1^ and Coulombic efficiency of 74.7%. Compared to the cell with the LLZO:LPSC = 8:2 composite electrolyte, the cell with the 7:3 composite electrolyte exhibited a remarkably enhanced specific capacity (Table 2). In addition, the Coulombic efficiency increased to 82.7%. These results are mainly attributable to the increase in the ionic conductivity of the composite electrolytes. The ionic conductivity of the LLZO:LPSC = 7:3 composite electrolyte is 3.5-times higher than that of the LLZO:LPSC = 8:2 composite electrolyte. This observation suggests that the ionic conductivity of the composite electrolyte plays a crucial role in enhancing the specific capacity of the ASSC.

Additionally, it was found that further improvement in the capacity of the LLZO:LPSC = 6:4 and 4:6 composite electrolyte cells was moderate, although the ionic conductivity increased with the LPSC ratio. This can be attributed to the contribution of the resistance due to the ionic conductivity of the composite electrolyte to the total resistance of the cell, which is relatively insignificant in the 6:4 and 4:6 composite electrolytes.

The charge–discharge curves of the cells with the composite electrolytes approximately matched the charge–discharge curve of the cell with the LPSC electrolyte, except for the parallel translation to the voltage axis, which is caused by the low ionic conductivity of the composite electrolytes. These results suggest that the NCM811 cathode/composite electrolyte interfacial contacts and ion-transport pathways were successfully formed in the composite electrolyte; thus, the performance of the composite-electrolyte-based cell was identical to that of the LPSC-electrolyte-based cell. Furthermore, the cycling performance of the ASSCs with the composite electrolytes at 0.1 C at 25 °C was investigated; the results are shown in Figure S1. Despite the fluctuations, the specific capacity of the cells with the LLZO:LPSC = 7:3 and 6:4 composite electrolytes was maintained at ~148 and ~158 mAh·g^−1^, respectively, and negligible capacity reduction was observed over 15 cycles. In addition, the ASSCs achieved over 99% Coulombic efficiencies. These results suggest that highly reversible lithiation and de-lithiation occurred in the ASSCs with the composite electrolytes.

## 4. Conclusions

Oxide (LLZO)/sulfide (LPSC) composite electrolytes and all-solid-state cells consisting of an NCM811 cathode, a composite electrolyte, and an In-Li alloy anode were fabricated by cold pressing. The cold-pressed LLZO electrolyte pellets were highly porous (39% porosity) and exhibited low ionic conductivity (~10^−7^ S·cm^−1^ at room temperature). The relative density measurements and SEM results indicate that the LLZO-LPSC composite electrolytes had a dense microstructure. The soft LPSC particles played a crucial role in improving the density of the composite electrolyte. Compared to the LLZO electrolyte, the composite electrolyte pellets exhibited ionic conductivities that were three orders of magnitude higher. Although the composite electrolytes of LLZO:LPSC = 8:2 and 7:3 featured a small semicircle in the impedance spectra, which was ascribed to the grain-boundary resistance, there was no semicircle in the spectra of the composite electrolytes of LLZO:LPSC = 6:4 and 4:6, indicating that lithium-ion conduction was governed by the sulfide electrolyte particles. The ASSC with the LLZO:LPSC = 7:3 composite electrolyte exhibited high capacity of 163 mAh·g^−1^ and Coulombic efficiency of 83%. The charge–discharge curves of the ASSCs containing the composite electrolytes with different LLZO:LPSC ratios confirmed that the NCM811 cathode/composite electrolyte interfacial contacts and ion-transport pathways were successfully formed in the composite electrolyte.

## Figures and Tables

**Table 1:** Room temperature (25 °C) ionic conductivities of LLZO, LLZO-LPSC composite, and LPSC electrolytes.

LLZO:LPSC (wt.%)	Ionic Conductivity (S·cm^−1^)
10:0	1.40 × 10^−7^
8:2	8.00 × 10^−5^
7:3	2.81 × 10^−4^
6:4	5.14 × 10^−4^
4:6	1.27 × 10^−3^
3:7 (LLZO:LPS)	5.4 × 10^−4^ [15]
3.3:6.7 (halide:LPS)	~10^−^^3^ [16]
0:10	2.92 × 10^−3^

**Table 2:** Specific capacities of ASSCs with composite electrolytes with different LLZO:LPSC ratios.

LLZO:LPSC (wt.%)	Specific Capacity (mAh·g^−1^)	
	Charge	Discharge
8:2	158	118
7:3	197	163
6:4	197	167
4:6	206	170
0:10	208	175

## Data Availability

Data sharing not applicable.

## Supplementary Materials

The following are available online at https://www.mdpi.com/article/10.3390/ma14081998/s1, Figure S1: Cycling performance of all-solid-state cells with composite electrolytes of LLZO:LPSC = 7:3 and 6:4.
