# Supplementary material for: All-Solid-State Lithium-Ion Batteries with Oxide/Sulfide Composite Electrolytes
Source: Materials (Basel). 2021 Apr 16;14(8):1998. doi: 10.3390/ma14081998 (PMC8073507; doi:10.3390/ma14081998)
Supplement: Supplementary file 1 [file materials-14-01998-s001.zip › materials-1166757-supplementary.pdf]

materials

Supplementary Materials

All-Solid-State Lithium-Ion Batteries with Oxide/Sulfide Composite Electrolytes

Young Seon Park [1], Jae Min Lee [1], Eun Jeong Yi [1], Ji-Woong Moon [2] and Haejin Hwang [1,*]

[1] Department of Materials Science & Engineering, Inha University, 22212 Incheon, Korea; pys3621@inha.edu (Y.S.P.); 22201275@inha.edu (J.M.L.); inha0326@inha.ac.kr (E.J.Y.)
[2] Battery Materials Research Center, Research Institute of Industrial Science and Technology, 37673 Pohang, Korea; jwmoon@rist.re.kr
* Correspondence: hjhwang@inha.ac.kr

Citation: Park, Y.S.; Lee, J.M.; Yi, E.J.; Moon, J.-W.; Hwang, H. All-Solid-State Lithium-Ion Batteries with Oxide/Sulfide Composite Electrolytes. *Materials* **2021**, *14*, 1998. https://doi.org/10.3390/ma14081998

Academic Editor: Alvaro Caballero

Received: 17 March 2021
Accepted: 13 April 2021
Published: 16 April 2021

Publisher's Note: MDPI stays neutral with regard to jurisdictional claims in published maps and institutional affiliations.

Copyright: © 2021 by the authors. Submitted for possible open access publication under the terms and conditions of the Creative Commons Attribution (CC BY) license (http://creativecommons.org/licenses/by/4.0/).

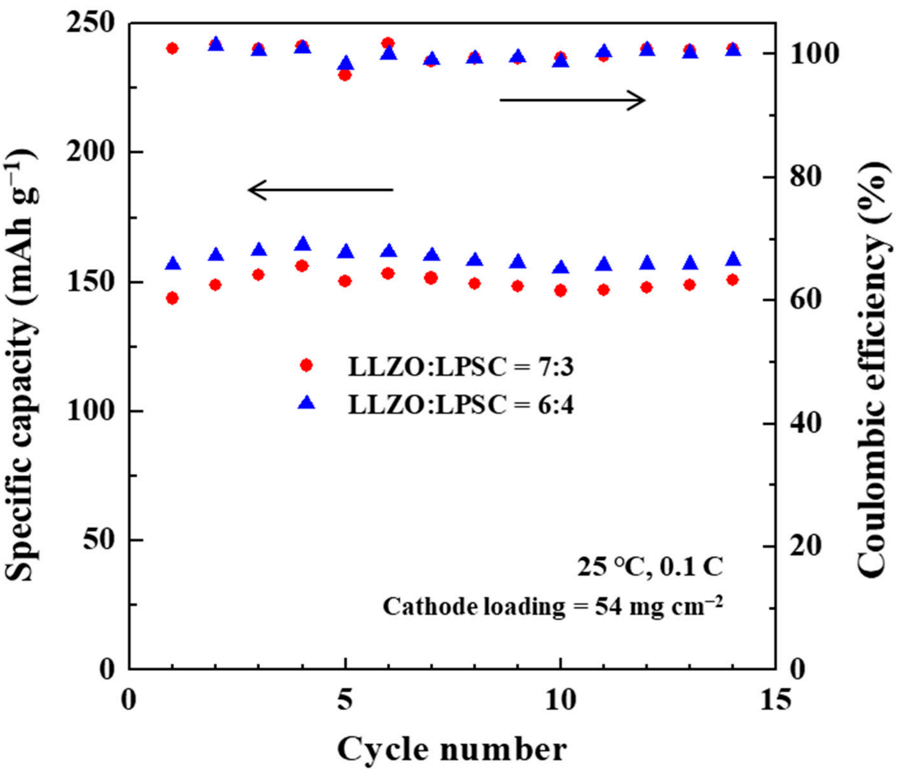

Figure S1. Cycling performance of all-solid-state cells with composite electrolytes of LLZO:LPSC = 7:3 and 6:4.
